# Protease Inhibition Improves Healing of The Vaginal Wall after Obstetrical Injury: Results from a Preclinical Animal Model

**DOI:** 10.1038/s41598-020-63031-6

**Published:** 2020-04-14

**Authors:** Jennifer Hamner, Maria Florian-Rodriguez, Jesus Acevedo, Haolin Shi, R. Ann Word

**Affiliations:** 1 Department of Obstetrics and Gynecology, Division of Female Pelvic Medicine and Reconstructive Surgery, University of Texas Southwestern Medical Center, Dallas, Texas USA; 2 Department of Obstetrics and Gynecology, Cecil H and Ida Green Center for Reproductive Biological Sciences, Division of Female Pelvic Medicine and Reconstructive Surgery, University of Texas Southwestern Medical Center, Dallas, Texas USA

**Keywords:** Physiology, Translational research

## Abstract

Vaginal delivery with obstetrical trauma is a risk factor for pelvic organ prolapse later in life. Loss of fibulin-5 (FBLN5), an elastogenesis-promoting cellular matrix protein, results in prolapse in mice. Here, we evaluated effects of pregnancy, parturition, and obstetrical injury on FBLN5 content, elastic fibers, biomechanics, and histomorphology of the vaginal wall in rats. Further, we analyzed the effects of actinonin, a protease inhibitor, on obstetrical injury of the vaginal wall. Vaginal FBLN5 decreased significantly in pregnancy, and injury resulted in further downregulation. Stiffness of the vaginal wall decreased 82% in pregnant rats and 74% (p = 0.019) with injury relative to uninjured vaginal delivery controls at 3d. Actinonin ameliorated loss of FBLN5, rescued injury-induced loss of elastic fibers and biomechanical properties after parturition, and reduced the area of injury 10-fold. We conclude that pregnancy and parturition have a profound impact on vaginal FBLN5 and biomechanics of the vaginal wall. Further, obstetrical injury has significant deleterious impact on recovery of the vaginal wall from pregnancy. Actinonin, a non-specific matrix metalloprotease inhibitor, improved recovery of the parturient vaginal wall after obstetrical injury.

## Introduction

Pregnancy and vaginal delivery are known independent risk factors for development of pelvic organ prolapse (POP)^1,2^. Literature is conflicting as to whether second-degree, or greater, obstetrical lacerations contribute to development of symptomatic POP^3,4^. Nonetheless, operative vaginal deliveries, which have a higher risk of obstetrical laceration, are known to confer higher risk for POP than spontaneous vaginal delivery^5^. As more data accumulate regarding poor long term outcomes from POP surgical interventions^6,7^. understanding the pathogenesis of prolapse and recovery of the pelvic floor after vaginal delivery are important in developing preventative measures.

The vaginal wall is comprised of epithelium, the subepithelial lamina propria, smooth muscle (muscularis), and adventitia. Vaginal extracellular matrix is composed of collagen and elastin fibers and smooth muscle^1^. Dysfunction or disruption of these components and/or the skeletal muscle structure of the pelvic floor have been linked with POP development^8^. Limited data exist regarding pregnancy and parturition-induced changes in the vaginal wall matrix and its mechanical properties. Knockout of Fibulin-5 (FBLN5), a matricellular glycoprotein protein that promotes elastogenesis in the female reproductive tract^9,10^, results in prolapse in mice^11,12^. Drewes *et al*. demonstrated an eightfold decrease in vaginal FBLN5 during pregnancy in mice^8^. Further, biomechanical properties of the vagina in pregnant mice included increased distensibility and decreased stiffness relative the vaginal wall of nonpregnant animals^13^.

The deleterious effects of pregnancy and vaginal delivery on the vaginal matrix have been previously linked to decreased expression of FBLN5 and concomitant upregulation of MMP-9 (a matrix degrading protein)^14^. Florian-Rodriguez *et al*. reported that actinonin, a protease inhibitor, blocked injury-induced degradation of FBLN5 and improved biomechanical properties of the vaginal wall after surgical injury in nonpregnant animals^15^. The potential role of protease inhibition on recovery of the vaginal wall after parturition or obstetrical injury is not known.

Given the limited understanding of the mechanisms by which pregnancy, parturition and obstetrical laceration impact vaginal recovery and or development of POP, the objectives of this study were to evaluate postpartum recovery of the vaginal wall with and without obstetrical injury using an animal model. Further, we sought to quantify effects of the protease inhibitor actinonin on vaginal FBLN5 content, elastic fibers, collagen content, biomechanics and healing of the parturient injured vagina.

## Materials and Methods

All animals were handled and euthanized in accordance with the standards of humane animal care described by the National Institutes of Health Guide for the Care and Use of Laboratory Animals, using protocols approved by the Institutional Animal Care and Use of Laboratory Committee (IACUC) of the University of Texas Southwestern Medical Center at Dallas. A total of 166 female Sprague Dawley rats (Charles River Laboratories) were housed in IACUC-approved facilities under a 12-L:12-D cycle at 22 °C. To obtain timed-pregnant animals, nulliparous females at 6–7 mos of age were housed with males overnight and checked for vaginal plugs in the morning. Plug day was considered day 0. Birth occurred late morning (after 10 am) of day 22 until midday on day 23. Rats were sacrificed as follows: nonpregnant (n = 6), late pregnant (day 21, n = 13), parturient (in labor after delivery of the first pup, n = 11), or postpartum time points (4 hours, n = 13, 12 hours, n = 15, 24 hours, n = 27, 3 days, n = 39, 7 days, n = 38, and 4 weeks, n = 12). Dams were allowed to lactate with pups until sacrifice. Maternal body weight at time of euthanasia for pregnant rats increased from 29.2 ± 1.0 g (nonpregnant) to 31.4 ± 2.6 g (pregnant day 21). Postpartum animals weighed 24.7 ± 0.3 g over all time points.

### Treatment groups

**Figure 1:**
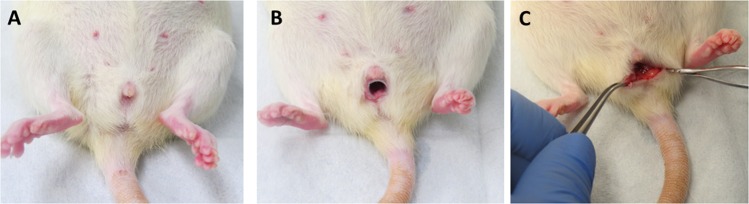
Surgical procedures. Late pregnant or newly parturient pregnant rats were anesthetized (**A**) and a modified speculum (**B**) was placed to visualize and generate laceration of the posterior vaginal wall extending from the posterior perineum to just below the cervix (**C**).

To facilitate timing of the injury, animals underwent simulated obstetrical laceration on the morning of gestation day 22 with delivery occurring within 28 h. Simulated obstetrical injury was created by a posterior vaginal wall incision from the perineum to the mid vagina through the epithelium and stroma (Fig. 1). At the time of injury, one dose of 200 µl actinonin (0.5 mg/mL) (Cayman Chemical, Ann Arbor, Mich) or phosphate buffered saline (PBS, 200 µl) was injected throughout the posterior wall injury using a 25 ga needle (100 µl on each side of the incision). Treatment groups were (1) normal vaginal delivery without injury, (2) injury + PBS injection, (3) injury + actinonin injection. Four animals were also treated with actinonin alone (without injury).

### Tissue processing

After euthanasia, the abdominal cavity was opened, the pubic symphysis disarticulated, and uterine horns, bladder, cervix, and vagina dissected down to the perineal skin. Using microsurgical instruments and a dissection microscope, the perineal skin was removed and the bladder and urethra separated from the anterior vaginal wall {Drewes, 2007 #1039}. Uterine horns and cervix were removed from the vagina. For animals randomized for histological analysis and biomechanical testing two rings were collected from the lower vagina. The vagina of animals used for immunoblotting was opened longitudinally (Fig. 1), epithelium scraped off with a scalpel, and the posterior vaginal muscularis (within 5 mm of injury) collected, snap frozen in liquid nitrogen and stored at −80 °C. Epithelium was removed since FBLN5 is a matrix protein.

### Matrix protein extraction

Frozen vaginal tissue was pulverized with a liquid nitrogen-chilled mortar and pestle as previously described {Drewes, 2007 #1039}. Tissue powder was then homogenized in basic buffer containing protease inhibitors (16 mM potassium phosphate, pH 7.8, 0.12 M NaCl, 1 mM ethylenediaminetetraacetic acid, 0.1 mM phenylmethylsulfonyl fluoride, 10 μg/ml pepstatin A, and 10 μg/ml leupeptin) and centrifuged at 10 000 × *g*. The supernatant was then removed, and the previous homogenization step repeated after resuspending the remaining tissue pellet in basic buffer. After removal of the second supernatant, the remaining tissue pellet was suspended in 6.0 M urea in the above basic buffer, homogenized, and placed on a rotating rack for overnight extraction at 4 °C. The samples were then centrifuged (10,000× *g* for 30 min), and the supernatant removed. Protein concentrations were determined using a bicinchoninic acid protein assay with standard curves of bovine serum albumin in appropriate buffers.

### Immunoblot analysis

Urea-extracted protein samples (15 μg/lane) were applied to 4%–20% gradient polyacrylamide gels (Bio-Rad), separated by electrophoresis, and transferred to polyvinylidene fluoride membrane {Florian-Rodriguez, 2019 #2322}. Identical gels were run side-by-side and Amido black-stained for protein loading comparison among the samples. After protein transfer, membranes were treated with blocking buffer, tris (hydroxymethyl) aminomethane 0.01 M, NaCl 0.15 M, Tween 20, 0.1%, pH 7.4 (TBS-T) with 2.5% nonfat milk for 1 h. The primary antibody for these studies (AB808) was generated by Thermo Fisher Scientific (Waltham, MA) by immunizing rabbits with the peptide ^76^YRGPYSNPYSTSYSGPYPAAAP^97^ of mouse and rat FBLN5. Sera were affinity purified and the antibody was shown to recognize a 65 kDa protein in mouse and rat vagina and aorta that is absent in tissues from FBLN5 knockout mice. Membranes were incubated in rabbit anti-rat FBLN5 (AB0809) at 1:500 dilution, overnight at 4 °C and then serially washed with TBS-T, followed by treatment with secondary antibody (goat immunoglobulin G anti-rabbit, 1:10,000) at room temperature for 1 h. Membranes were serially washed with TBS-T and subsequently incubated with Supersignal West Pico PLUS (Thermo Fisher Scientific) for 2 min. Signal strength was captured using the ChemiDoc XRS + (Bio-Rad, Hercules, CA) image capture system. Protein band volume was calculated using Image Lab version 6.0 software (Bio-Rad, Hercules, CA) and normalized to total protein loaded quantified on amido black stained gels.

### Biomechanical testing

Vaginal rings from the distal vagina (~1–2 mm thick) were suspended between two stainless steel wire mounts and attached to a steel rod apparatus with a calibrated mechanical drive and to a force transducer. Tissues were maintained in calcium-containing physiologic salt solution in water baths at 37 °C with 95% O_2_ and 5% CO_2_ as described previously {Rahn, 2008 #1575}. After acclimation for 15 min, each ring was equilibrated to slack length through a preconditioning protocol of serial stretches that returned to baseline tone. Ring diameter was measured at resting tone by the calibrated mechanical drive. Rings were distended in 1 mm increments with 30-sec intervals between each increment to allow stabilization of forces before each subsequent distention. This process was continued until failure (ring breakage) or until plateau of force generation. Wet weights of vaginal rings were determined after testing. Stress (kPa) was calculated as maximum force per unit cross-sectional area and plotted against strain (change in length divided by slack length), producing a sigmoid-shaped curve. Cross-sectional area was computed as described previously^13^. Tissue stiffness was calculated from the slope of the linear portion of the curve.

### Histomorphology

Vaginal rings were fixed in neutral buffer formalin (10%). Tissues were subsequently processed and embedded in paraffin blocks. Cross-sections of each vaginal ring (4 µm) were stained with Masson trichrome and Hart’s stain using standard technique. Images of each Masson trichrome section were captured and analyzed using a Nikon E1600 microscope and Nikon NIS Elements AR software (Melville, NY). ImageJ 1.52 software (NIH, Bethesda, MD) using a default threshold of “MaxEntropy” was used to analyze elastin tissue composition in the vaginal muscularis of Hart’s stained sections.

### Immunohistochemistry

Slides were deparaffinized and rehydrated for immunohistochemistry analysis. Rehydration occurred by immersing slides in Xylene and graded ethanols. Antigen retrieval was performed using a 10 mM sodium citrate buffer (pH 6.0) for 30 minutes. Sections were then incubated overnight with both collagen I and III primary antibodies after blocking for 30 minutes with 10% normal goat serum (Thermo Fisher Scientific, Walthan, MA). Rabbit anticollagen I (1:200) (Abcam, Cambridge, MA) and mouse anticollagen III (1:500) (Sigma-Aldrich, St. Louis, MO). The optimal dilutions of each antibody were determined by multiple titration experiments. Secondary antibodies for Collagen I and III, goat antirabbit Alexa Flour 568 (1:500)(Abcam, Cambridge, MA) and goat antimouse Alexa Flour 488 (1:500, Thermo Fisher Scientific, Walthan, MA) respectively, were incubated for 1 hour followed by 2 washes of phosphate-buffered saline. ProLong Gold antifade reagent with DAPI (Invitrogen, Eugene, OR) was placed with coverslip and dried in the dark overnight at 4 °C. Slides were scanned using Confocal Zeiss LSM880 Airyscan (Jena, Germany) with a 20x objective. A single fluorescent image of the vaginal muscularis from each slide (at the site of injury if present) was analyzed by a single author (JH). Analysis was performed using the ImageJ 1.52 software (NIH, Bethesda, MD) using a default threshold of “Moments” for quantification. Results were analyzed as area fraction (percentage of pixels) of each collagen subtype in vaginal muscularis, excluding blood vessels and epithelium.

### Statistical analysis

Since biomechanical parameters are the most variable of endpoints in this study, the power analysis was based on data from biomechanical parameters conducted previously, requiring 6 in all groups for 80% power to detect a difference at p < 0.05. Additional rats were included to allow for potential loss of animals or problems with tissue handling/dissection. Analysis of variance (ANOVA) testing was performed for multiple group comparisons using Sigmastat software (Jandel Scientific, San Rafael, CA). Post testing between groups was performed using Holm-Sidak method for normally distributed data and Dunn tests for nonparametric distributions. Comparisons between two groups as a function of time were by 2-way ANOVA. Student’s t test was used for comparison of two independent groups. P < 0.05 determined significance.

## Results

### FBLN5 content in the vaginal wall of antepartum and postpartum rats

**Figure 2:**
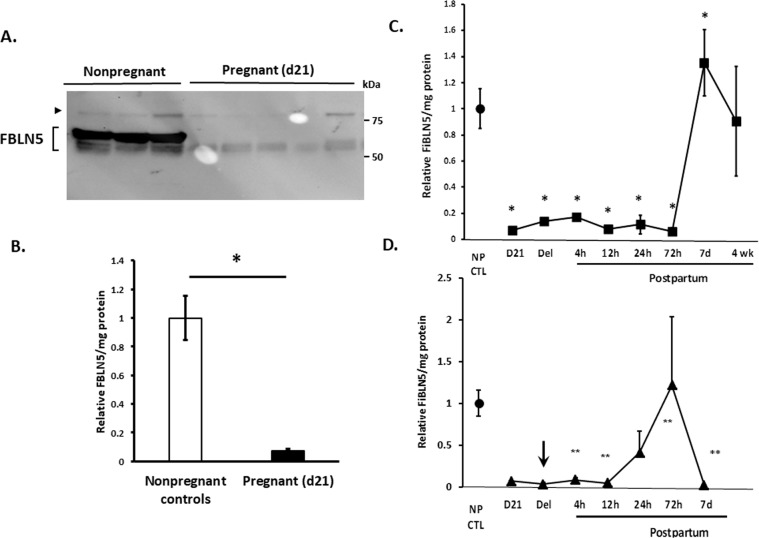
Effect of pregnancy and normal parturition on vaginal FBLN5. (**A**) Representative immunoblot (30 µg urea-extracted protein per lane) of vaginal muscularis from nonpregnant controls and late pregnant animals. The >75 kDa protein is nonspecific interaction of protein with primary antibody. Specific FBLN5 immunoreactive proteins are noted by brackets with proteins of lower molecular weight likely representing differences in glycosylation or splice variants. Bands within brackets were combined for quantification. (**B**) Quantification of FBLN5 per mg protein in vaginal muscularis from nonpregnant (n = 6, **NP CTL**) and late pregnant (n = 13) rats. *P < 0.01, Student’s t test. (**C**) Effect of pregnancy and parturition without injury on FBLN5 content of the rat vaginal wall. Data represent mean ± SEM of relative FBLN5/mg protein in 4 animals per group. *One-way analysis of variance (P < 0.05). (**D**) Effect of simulated obstetrical injury on recovery of FBLN5 content in the postpartum vaginal wall. Arrow denotes time of injury as described in Materials and Methods. **significant differences relative to noninjured time point in Panel **C**. Data represent mean ± SEM of 4 animals in each group.

Vaginal FBLN5 in pregnant (day 21), parturient, and postpartum animals (4 h, 12 h, 24 h, 3d, 7d, 4w) was quantified by immunoblot analysis of urea-extracted muscularis, normalized to total matrix protein by amido black staining, and expressed relative to nulliparous nonpregnant controls (served as standards on each gel). Vaginal FBLN5 was decreased 93% in pregnant (day 21) animals relative to nonpregnant controls (Fig. 2A,B). In contrast to the reported surge in FBLN5 4–24 h postpartum in mice, FBLN5 expression remained suppressed from pregnancy (day 21) through the 3rd postpartum day in rats. FBLN5 levels surged 7d postpartum with values modestly, but significantly, increased relative to nonpregnant controls. Thereafter, levels returned to nonpregnant levels by 4 weeks (Fig. 2C).

### Effect of simulated obstetrical injury on postpartum recovery of vaginal FBLN5

Next, we sought to determine if simulated obstetrical injury affected recovery of vaginal FBLN5 after parturition. To facilitate timing of the injury, animals underwent simulated obstetrical laceration on the morning of gestation day 22 and delivery occurred within 28 h. Compared with spontaneous vaginal delivery without injury in which vaginal FBLN5 was already suppressed (Fig. 2C), FBLN5 was decreased even further after injury (Fig. 2D). Importantly, the time course of recovery after injury differed significantly compared with recovery without injury. Specifically, injury resulted in modest increases in FBLN5 24 h − 3d after injury relative to normal delivery in which FBLN5 remained suppressed through the 3d time point. Nonetheless, this modest partial response to injury was depleted by 7d such that vaginal FBLN5 was barely detectable and strikingly decreased relative to recovered levels 7 d postpartum without injury. Thus, injury prevented the normal robust recovery of vaginal FBLN5 7d postpartum.

### Actinonin at time of vaginal injury alters FBLN5 content in the postpartum vaginal wall

**Figure 3:**
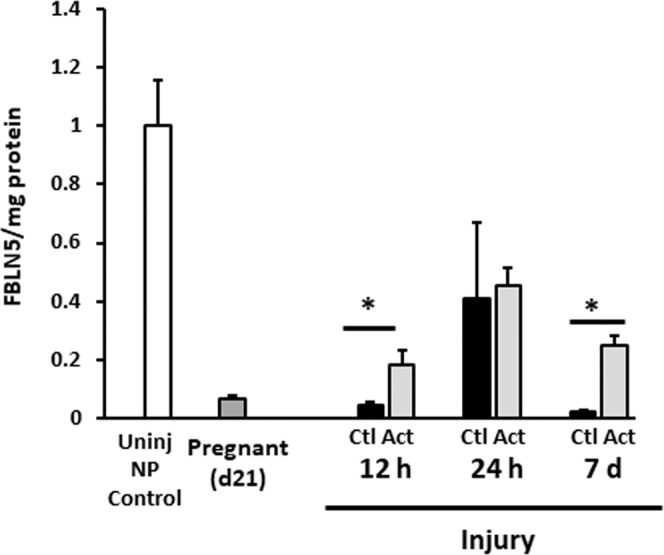
Effect of actinonin at time of simulated obstetrical injury on FBLN5 content in the rat vaginal wall. Relative FBLN5/mg protein in postpartum animals after injury near time of delivery. Data represent mean ± SEM of 4 animals in each group. **Uninj NP Control**, nonpregnant control without injury (open bar); Pregnant (21), dark grey; **Ctl**, injury + PBS, solid bars; **Act**, injury + actinonin,light grey; **NP**, nonpregnant. *P < 0.05, two-way analysis of variance.

Previously, we showed that a broad-spectrum MMP inhibitor (actinonin) blocked injury-mediated degradation of vaginal FBLN5 in nonpregnant rats after surgical injury^15^. Having established low levels of FBLN5 protein in the parturient vagina, we quantified the effects of actinonin on recovery of FBLN5 after delivery (Fig. 3). Pregnant rats at day 22 of gestation (prior to delivery) underwent posterior vaginal wall injury, and were injected locally with either PBS (control) or actinonin (0.5 mg/mL) at the time of injury. For these experiments, epithelium was removed with a scalpel and the remaining lamina propria and muscularis underwent matrix extraction. As shown in Figs. 2D and 3, FBLN5 was downregulated 95 ± 0.8% (12 h), 59 ± 25% (24 h) and 98 ± 0.9% (7d) in control injured postpartum rats from nonpregnant levels (p =< 0.001, p = 0.021, p = 0.021, Fig. 3). Actinonin administration at the time of injury ameliorated injury- and delivery-induced loss of vaginal FBLN5 (12 h, p = 0.021; 7d, p =< 0.001, Fig. 3). FBLN5 levels, however, were only partially rescued by actinonin.

### Effect of pregnancy and postpartum +/− injury on vaginal wall elastin content

**Figure 4:**
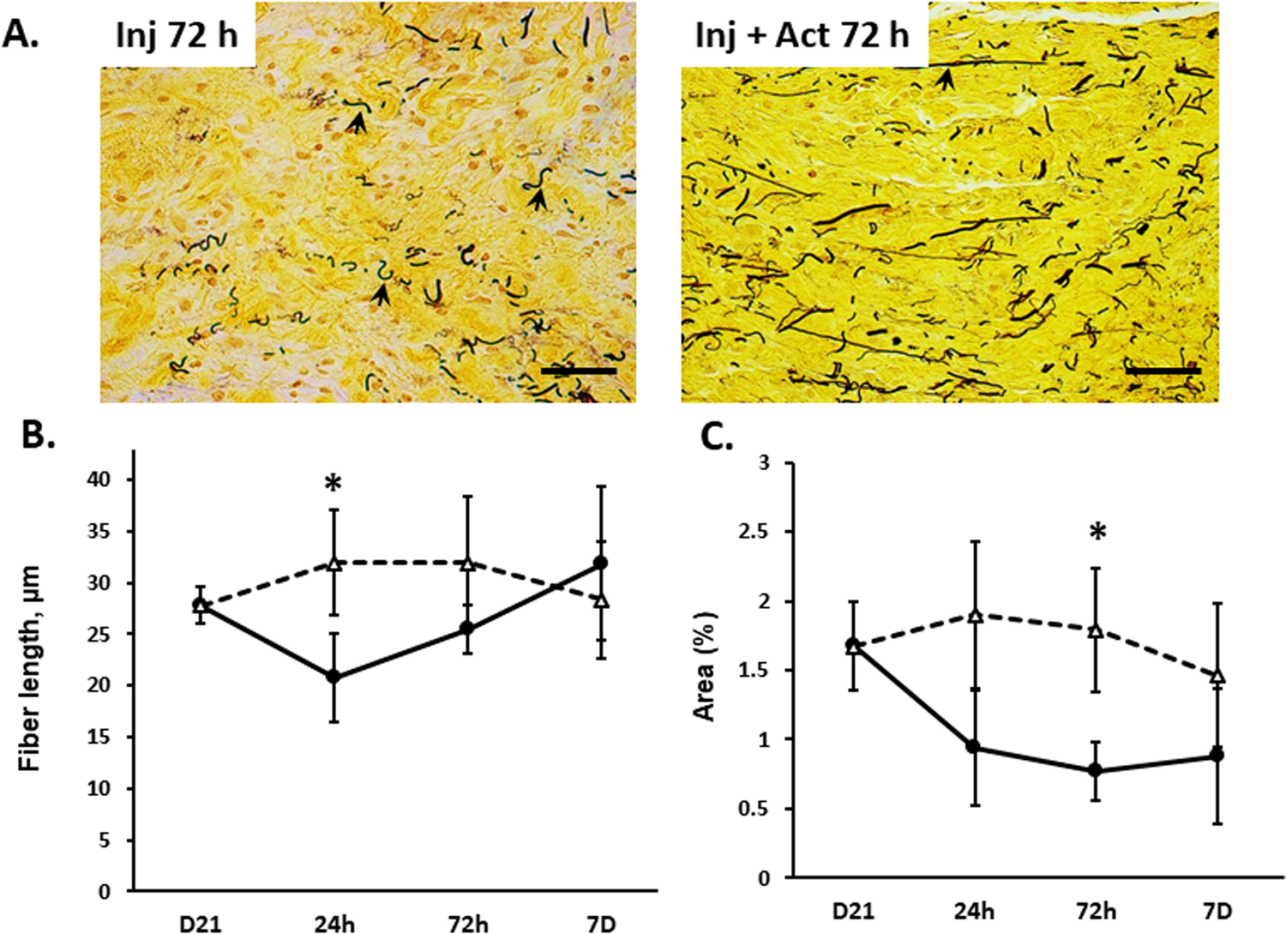
Effect of injury on vaginal elastic fibers with injury ± actinonin. Microscopic images of vaginal muscularis on 3d postpartum in (**A**) injury + PBS (**Inj 3d**) and injury + actinonin (**Inj** + **Act 3d**). The 4–5 µm sections of full-thickness vaginal rings were stained with Hart’s stain. Quantitative analysis performed using a Nikon E1600 microscope with 40x objective and ImageJ software. Scale bars 50 µm. Graphical depiction represents (**B**) elastin fiber length (µm) and (**C**) percent area covered by elastic fibers in pregnant (**D21**) controls compared to postpartum time points for injury with (triangles) and without actinonin (solid circles). n = 4–5 in all groups, *P < 0.05 compared with injury alone time point, t-test.

Downregulation of the elastic fiber organizer, vaginal FBLN5, during pregnancy and the early postpartum time period led us to investigate the effect of vaginal delivery, injury, and injury + actinonin on vaginal elastic fibers. Pregnant rats (day 21), and 24 h, 3d, 7d, and 4w postpartum time points with or without injury (+/- actinonin) were collected for Hart’s staining of elastic fibers in the posterior vaginal wall. Histologic analysis was performed and elastic fibers expressed as mean fiber length and % cross sectional area of the subepithelium and muscularis layer only (Fig. 4 and Supplemental Fig. 1). During pregnancy and the postpartum time period, elastic fibers were thin and long, covering 1.7% of the total cross sectional area. Elastic fiber length and % cross sectional area were stable throughout the uninjured postpartum time period (Supplemental Fig. 1C,D). In contrast, in 3 of 5 injured samples, elastic fibers were disrupted in the injured vagina and highly localized to the area of injury (Fig. 4A). Fiber length decreased within 24 h of injury relative to normal vaginal delivery (20.7 ± 4 compared with 36 ± 8 µm, p < 0.05, compare Supplemental Fig. 1 with Fig. 4) and did not recovery substantially by 3d (Fig. 4). In general, elastic fiber area decreased 3d after injury (i.e., ≤ 1% in 4 samples, but normal in 1). Notably, actinonin rescued injury-induced loss of elastic fiber length at 24 h and fiber area at 3d (Fig. 4B,C). Actinonin was not universally effective. Recovery of elastic fiber area was quite remarkable in 3 of 5 samples (to 2.3, 2.8, and 2.4%) but remained compromised in 2 of 5 samples. Thus, fiber morphology was only partially restored with short thicker fibers remaining even after actinonin treatment (Fig. 4). Together, the data indicate that elastic fiber length and area do not change appreciably in the rat vagina after vaginal delivery. Injury, however, results in localized loss of elastic fibers which is partially rescued with actinonin.

### Histomorphology of the vaginal wall during pregnancy and postpartum period with or without injury

**Figure 5:**
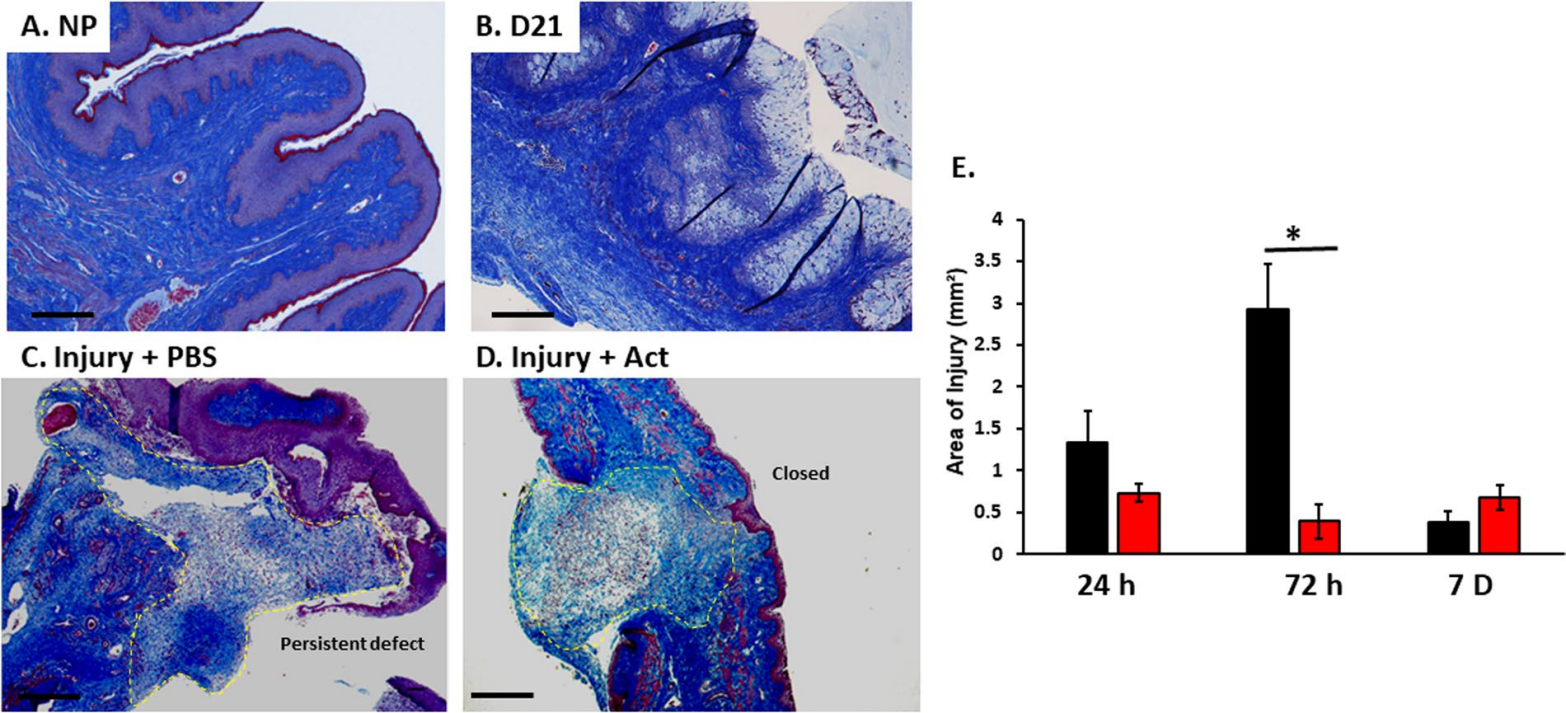
Representative histologic images of nonpregnant, day 21 and postpartum vaginal wall after injury. Microscopic images of vaginal muscularis and epithelium in nonpregnant (**NP**) (**A**), day 21 (**B**), 3d after delivery in injury + PBS (**C**) and injury + actinonin (**D**). The 4–5 µm sections of full-thickness vaginal rings were stained with Masson trichrome. Quantitative analysis of area of injury (outlined with yellow dashed line. Vaginal rings in the actinonin treated specimen (**D**) are intact with statistically smaller area of injury. Injury + PBS specimens (**C**) maintain persistent defects at injury site. (**E**) Area of injury (mm²) for postpartum injured animals treated with PBS (black) or actinonin (red). **PBS**, phosphate buffered saline; **Act**, actinonin. 4X magnification, Scale bar, 500 µm.

FBLN5 has been shown to serve a dual role in the vaginal wall. It is not only an elastic fiber organizer but also inhibits vaginal MMP9, which plays a major role in matrix remodeling after injury. To investigate the effect of pregnancy and vaginal delivery with or without injury on matrix homeostasis of the vaginal wall, trichrome stains were conducted and analyzed. Vaginal epithelial thickness increased from 66 ± 5 µm in nonpregnant animals to 129 ± 33 µm (p < 0.05) in late pregnant rats (Day 21) with gradual thinning of the epithelium to 66% of nonpregnant levels by 7d (p < 0.001)(Fig. 5A,B). Muscularis thickness, however, was similar between nonpregnant controls and pregnant animals at all time points (Fig. 5A,B).

Within 24 h after obstetrical injury, the wound was characterized by dramatic infiltration of neutrophils, monocytes, and macrophages at the area of injury measuring 1.3 ± 0.38 mm^2^ (Fig. 5C,E and Supplemental Fig. 2). Immune cell infiltration also occurred 24 h after normal delivery without injury (Supplemental Fig. 2B). In contrast to injury, however, with normal vaginal delivery the infiltrate was largely confined within blood vessels with modest infiltration into the stromal tissue. Notably, the matrix remained intact pericellular to the immune cells after normal vaginal delivery. The pericellular matrix was degraded surrounding immune cells of the injured vagina generating a “halo” effect (Supplemental Fig. 2C). By 3d, the area was even larger (2.9 ± 0.53 mm^2^) with an overt increased number of macrophages (Fig. 5C and Supplemental Fig. 2) and matrix degradation. Actinonin resulted in complete closure of the wound at 3d and reduced the area of injury almost 10-fold to 0.39 ± 0.19 mm^2^ (P < 0.004, Fig. 5D,E).

### Biomechanical properties of the vaginal wall during pregnancy and postpartum

**Figure 6:**
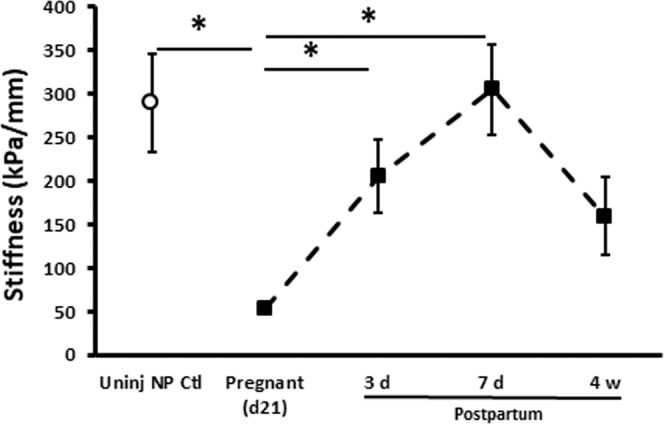
Effect of pregnancy and uninjured normal postpartum period on biomechanical properties of the rat vaginal wall with vaginal delivery. Stiffness (resistance to deformation, kPa/mm) was quantified from the slope of the linear portion of stress-strain curves. Data represent mean ± SEM of 8 animals in each group. *One-way analysis of variance (P < 0.05).

To determine if the effects of injury ± actinonin on FBLN5 and histomorphometry translated into functional effects on the vaginal wall, we quantified biomechanical properties of the vaginal wall during pregnancy and postpartum time period with and without injury. Consistent with prior studies in mice, the stress-strain curves of nonpregnant rats revealed marked increases in stress generation with small increases in strain, i.e. increased stiffness. On the other hand, stiffness (resistance to deformation) of the uninjured distal vagina from pregnant (day 21) animals was decreased 82% (p = 0.002, Fig. 6) facilitating increases in vaginal distention to 20.8 mm compared with 11.3 mm for nonpregnant animals (Table 1). Recovery of vaginal stiffness postpartum was biphasic with an initial return to non-pregnant levels by 7 days falling to levels less than virginal animals by 4 wks (Fig. 6).

**Table 1:** Biomechanical properties of the vaginal wall in nonpregnant controls, late pregnancy (d21), 3–7d after delivery without injury, 3–7d after delivery with injury + PBS, and 3–7d after delivery with injury + actinonin.

	Total Vaginal Weight, mg	Maximal distention mm	Resting diameter mm
**Nonpregnant**Controls, n = 7	159 ± 9	11.3 ± 1.6	9.6 ± 1.2
**Pregnant, D21** n = 7	201 ± 22^*^	20.8 ± 2.1*	10.2 ± 0.6
**Postpartum, 3d** Control, n = 7	142 ± 24	12.4 ± 1.1	9.3 ± 0.9
**Postpartum, 3d** Inj + PBS, n = 4	197 ± 13^*^	16.5 ± 2.4	6.8 ± 0.3
**Postpartum, 3d** Inj + Act, n = 6	168 ± 7	14.8 ± 0.5	6.8 ± 1.5
**Postpartum, 7d** Control, n = 7	140 ± 10	14.5 ± 1.0	8.8 ± 0.4
**Postpartum, 7d** Inj + PBS, n = 8	141 ± 5	14.7 ± 2.2	8.4 ± 1.2
**Postpartum, 7d** Inj + Act, n = 8	161 ± 13	16.9 ± 1.1	7.8 ± 0.6

Mean ± SEM

PBS, phosphate buffered saline; Inj, injury; Act, actinonin.

*p < 0.05 compared with NP and all postpartum time points.

**Figure 7:**
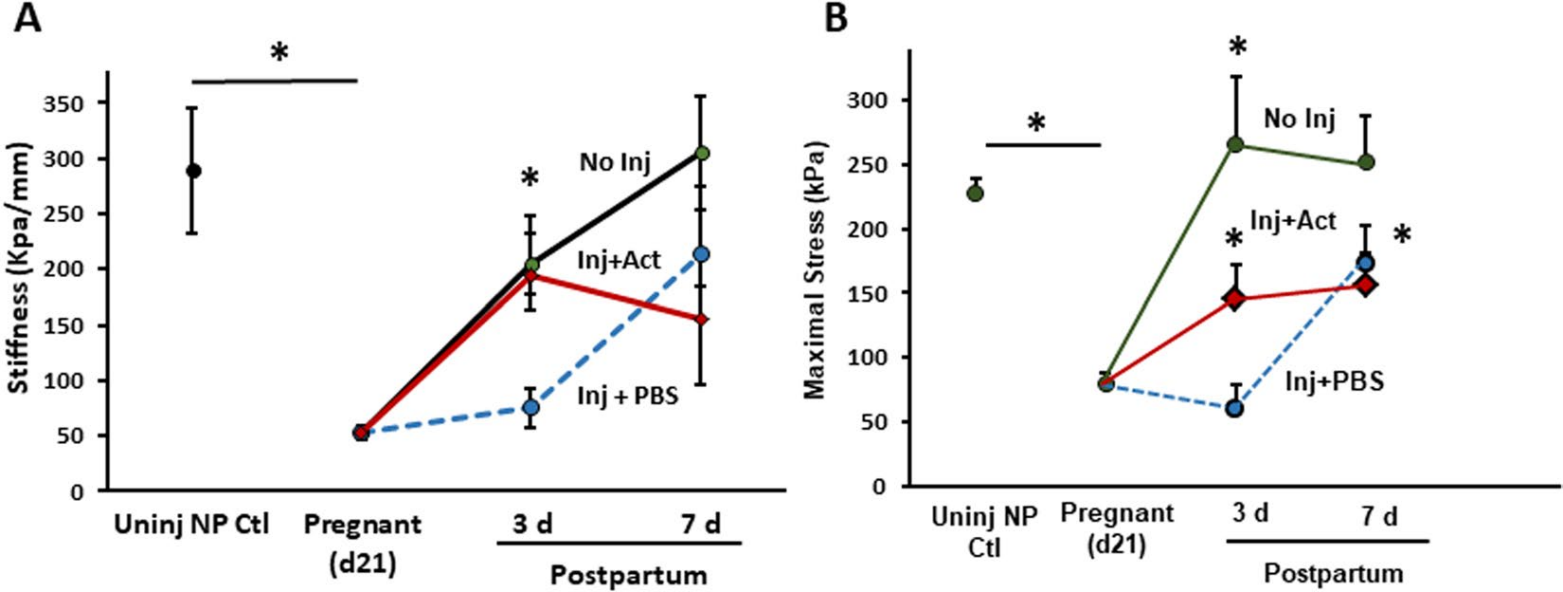
Effect of actinonin on biomechanical properties of the injured rat vaginal wall at the time of vaginal delivery. (A) Stiffness was quantified from the slope of the linear portion of stress-strain curve. Postpartum time points of 3 and 7d after injury are presented. (**B**) Maximal stress generating capacity in nonpregnant, pregnant, and postpartum animals with or without injury. Data represent mean ± SEM of nonpregnant (n = 8), normal postpartum (n = 8 per time point), injury + PBS (n = 4 at 3 d, n = 8 at 7 d) and injury + actinonin (n = 6 at 3d, n = 8 at 7d). For clarity, positive error bars are shown. Actinonin alone (without injury) did not alter normal recovery (Supplementary Fig. 3). **Inj**, injury; **Uninj**, uninjured; **NP**, nonpregnant; **Act**, actinonin; **PBS**, phosphate buffered saline. *Two-way analysis of variance (P < 0.05).

Next, we conducted biomechanical studies of the vagina from injured pregnant animals (Fig. 7 and Table 1). Injury had a significant impact on biomechanical recovery of the posterior distal vaginal wall. Specifically, vaginal stiffness decreased 74% (p = 0.019) 3d after injury relative to normal recovery (Fig. 7A). Maximal stress (an index of tissue strength) was also decreased after injury relative to normal postpartum 3d (Fig. 7B). Four of eight lacerated tissues did not close and biomechanics of the ring was not possible. Interestingly, actinonin rescued injury-induced loss of stiffness with near complete recovery at 3d (p = 0.014, Fig. 7A). Two of 8 tissues remained open at 3 d. Maximal stress also increased significantly with actinonin (p = 0.034, Fig. 7B). Actinonin alone at the time of normal vaginal delivery did not alter biomechanical properties of the postpartum vaginal wall.

### Effects of pregnancy and postpartum on collagen in the vaginal wall

**Figure 8:**
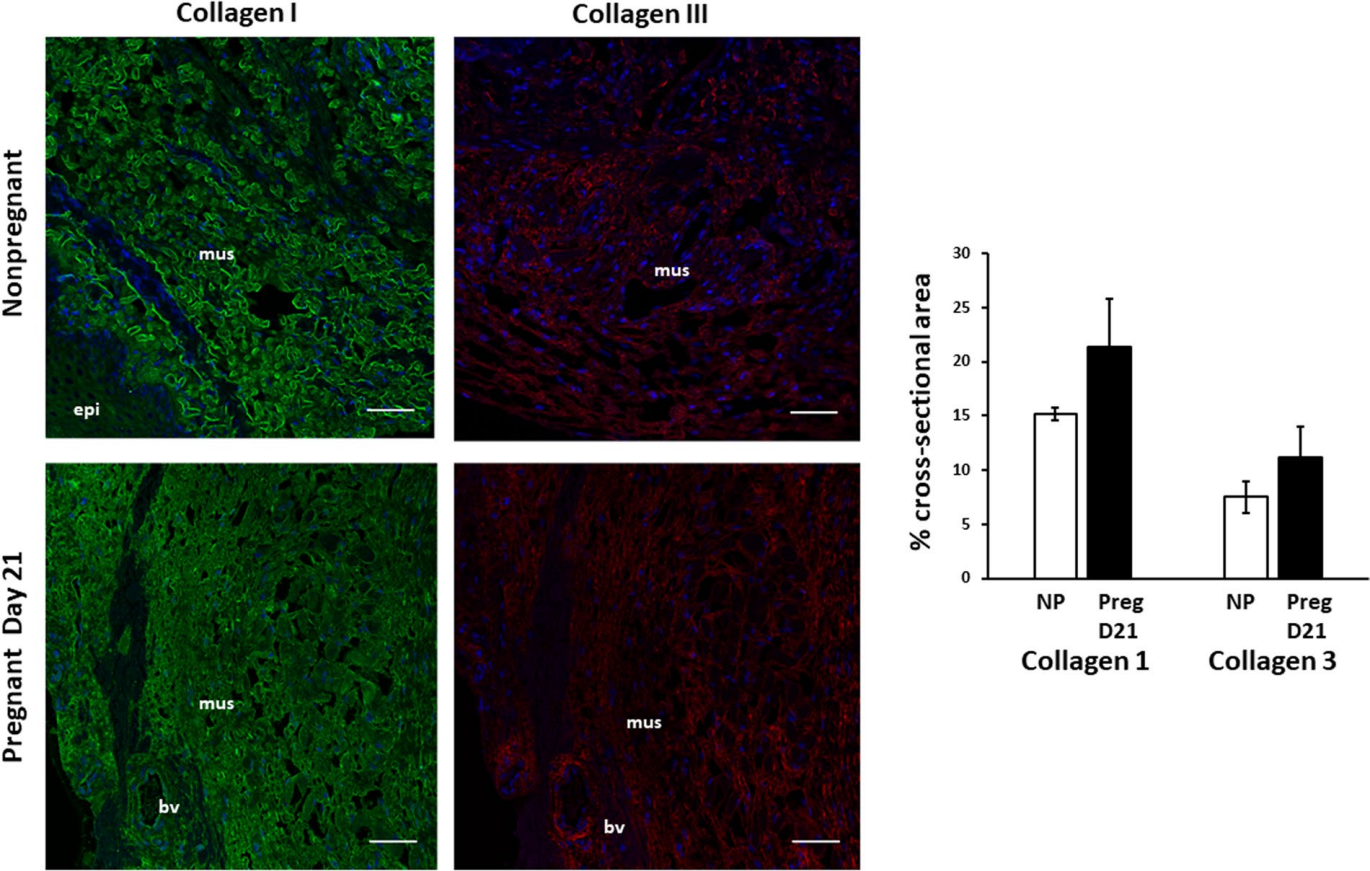
Immunolocalization of Collagen Types I and III in the vaginal wall of pregnant and nonpregnant rats. Immunofluorescence of Collagen I (*LEFT*) and III (*RIGHT*) in uninjured rat vaginal wall in nonpregnant virginal rats (top row) and pregnant rats on day 21 of gestation (bottom row). Sections of full-thickness vaginal rings (4–5 µm) were labeled with primary antibodies to collagen I and III. *Green* = collagen I, *Red* = Collagen III, *Blue* = DAPI. Increased ratio of collagen I/III noted in each specimen. Increased content of both collagen subtypes noted in pregnant specimens. Scale bars 50 µm. Graphical depiction (Right) of collagen area between nonpregnant controls (**NP**) and pregnant (**Preg D21**) animals. **mus**, vaginal muscularis; **epi**, epithelium**; bv**, blood vessel.

**Figure 9:**
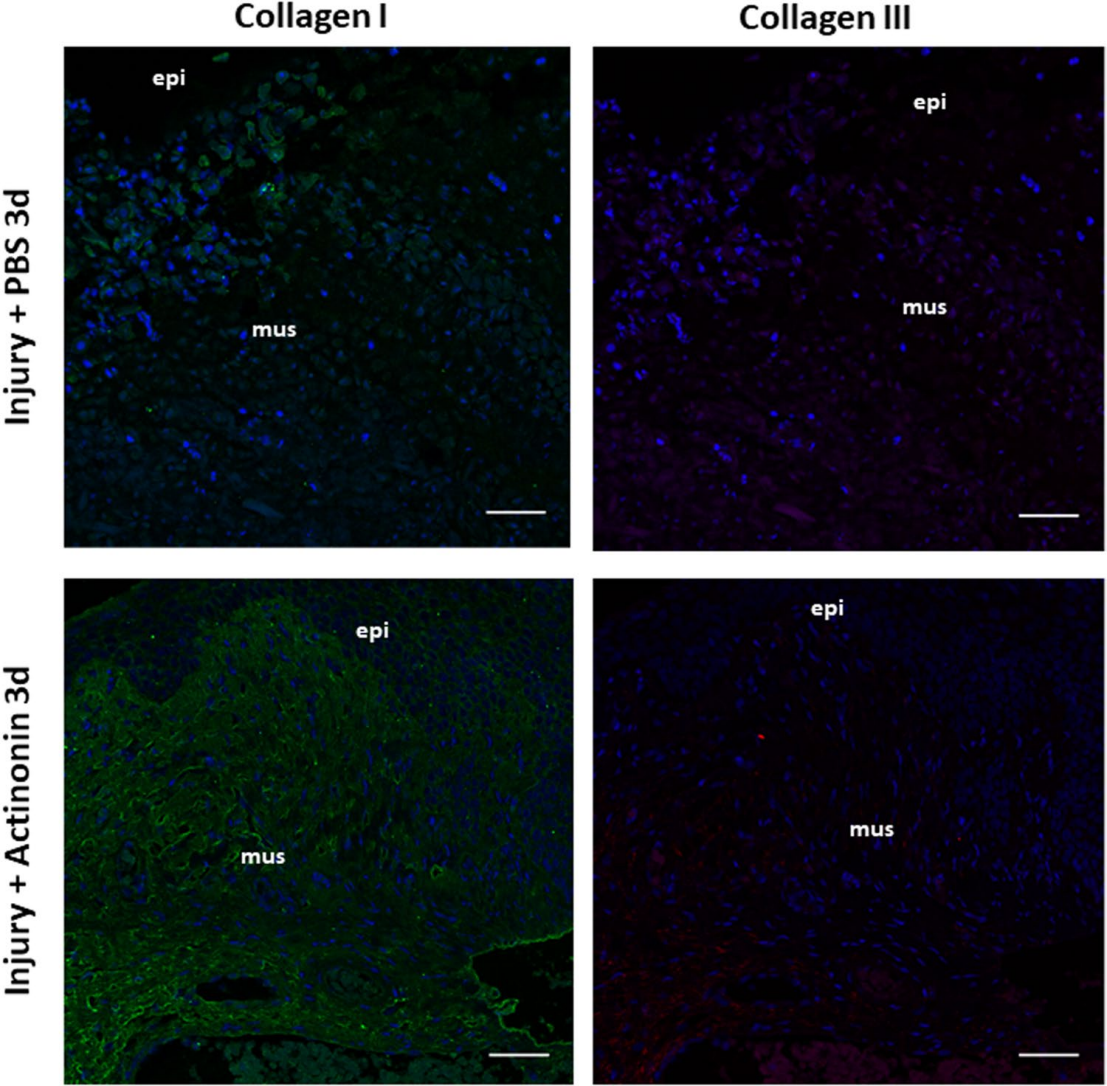
Immunolocalization of Collagen Types I and III in the vaginal wall after obstetrical injury. Immunofluorescence of Collagen I (1^st^ column) and Collagen III (2^nd^ column) in rat vaginal wall at site of injury 3 days after delivery in injury+ PBS (top row) and Injury+ actinonin (bottom row). *Green* = collagen I, *Red* = Collagen III, *Blue* = DAPI. Scale bars 50 µm. **PBS**, phosphate buffered saline; **Act**, actinonin; **mus**, vaginal muscularis; **epi**, epithelium.

We considered the possibility that differential expression or distribution of fibrillar collagen subtypes I and III may contribute to the altered biomechanical properties during pregnancy. Immunohistochemistry of collagen I and III and quantitative analysis using scanning confocal microscopy with a 20x objective and ImageJ software revealed that collagen type I was predominant relative to collagen type III. Ratios of subtypes did not change with pregnancy (Fig. 8). Both collagen types I and III were lost at the site of injury 3 d postpartum which were rescued with actinonin (Fig. 9).

## Discussion

The vaginal wall is suspended to the pelvic muscles and is believed to play an important role in pelvic organ prolapse^16,17^. Support of the pelvic floor includes a continuous sheet of connective tissue that envelops the vagina. Previously defined as “*endopelvic fascia*,” this connective tissue coalesces laterally to the arcus tendineous fascia pelvis and superior fascia of the levator ani. Since history of vaginal delivery is a primiary risk factor for POP, we reasoned that aberrations in physiologic remodeling or pathophysiologic injury of the vagina may contribute to the pathogenesis of prolapse later in life.

Parturition in rats is not as traumatic as childbirth in humansUsing a model of simulated obstetrical injury (posterior vaginal wall laceration), we found that obstetrical laceration impacted recovery of the parturient vaginal wall.

### Impact of obstetrical injury on recovery of the parturient vaginal wall

Animal models suggest that defects in elastic fibers lead to pelvic organ prolapse^8,14,18,19^. The protein FBLN5 (a) is required for assembly and organization of tropoelastin into mature elastic fibers^9^, (b) aids in localization of LOXL-1 onto elastic fibers^18^, and (c) is crucial to inhibit vaginal MMP9 activity^11^. The dual role of FBLN5 in elastogenesis and inhibition of vaginal MMP9 provides a strong link between elastic fiber assembly and collagen homeostasis. Previously, we reported that vaginal tropoelastin and FBLN5 decrease in late gestation and the early postpartum time period (2–4 h) in mice^8^. Here, we confirmed these findings in rats but FBLN5 was downregulated in the lamina propria and muscularis for longer times in the postpartum time period (>3d) compared with mice. In contrast with the mouse, elastic fibers appeared normal in the vagina throughout the puerperium in rats, but were vulnerable to injury at the time of delivery.

Our results indicate that recovery of the vaginal wall after normal parturition includes (i) return of vaginal FBLN5 to nonpregnant levels within 7d, (ii) return of compromised vaginal stiffness and strength by postpartum 7d, (iii) no change in elastic fiber length or area, (iv) transient limited trafficking of immune cells into the vagina, and (v) return to prepregnancy vaginal weight. The lack of change in elastic fiber density is consistent with previous reports using the same animal model. Downing *et al*. found that elastic fiber tortuosity was decreased 2d postpartum returning to normal 2 weeks postpartum^20^. Here, we did not evaluate tortuosity of the fibers quantitatively, but visual inspection is in agreement in that postpartum elastic fibers appeared straighter. Obstetrical injury amplified postpartum decreases in vaginal FBLN5 and delayed return to prepregnancy levels. Further, injury resulted in highly localized loss of elastic fibers and collagen types I and III at the site of injury. Finally, injury delayed recovery of vaginal biomechanical properties. This may be important as new mothers with changed body habitus return to exercise and physical activity which may impose increased forces on a compromised pelvic floor after injury^21^. It should be emphasized that biomechanical studies were conducted in Ca^2+^-containing solution which does not allow us to differentiate between contributions of smooth muscle cells vs other cellular and extracellular components.

During pregnancy, vaginal weight and collagen types I and III increase. Despite these increases in collagen content, stiffness decreased dramatically, an adaptation believed to facilitate distention of the vaginal wall during delivery. With injury, immunohistochemistry indicated overt loss of both collagen I and III and compromised biomechanical function of the vaginal wall. Thus, our histologic and biomechanical results are concordant indicating that localized loss of fibrillary collagens I and III occur with obstetrical injury and biomechanical recovery is delayed after parturition. The increase in collagen content with pregnancy accompanied by profound decreases in stiffness remains a paradox. Although collagen content, subtypes, and elastic fibers usually dictate biomechanical properties, other factors may be involved including collagen alignment and collagen dispersal by proteoglycans and hyaluronan which were not evaluated in this study.

Previous studies of the biomechanical adaptions of the rat vagina and supportive tissue due to pregnancy are in good agreement with findings reported herein. Specifically, linear stiffness of the vagina and support tissues was reduced in late-pregnancy and immediately after vaginal or abdominal delivery^22^. Similarly, studies regarding passive and active mechanics of the rat vagina revealed that the tangent modulus of pregnant animals was decreased returning to that of virgin rats within 4 weeks^23^. In the current study, we used a protocol that defines the biomechanical properties of vaginal tissues from the mid-lower vagina with or without injury at multiple postpartum time points. Although simulated obstetrical injury had a negative effect on postpartum recovery, biomechanical properties recovered partially by 7d. Currently, there is no clear definition of optimal vaginal biomechanical properties. A tissue that resists deformation (stiff) with increased tensile strength may represent fibrosis causing dyspareunia and failure to distend with pregnancy. On the other hand, it is necessary to decrease stiffness to allow for distention of the vaginal wall during pregnancy. Here, stiffness decreased dramatically during pregnancy requiring 7 days to near recovery at 7 d. After injury, recovery of pregnancy-induced loss of stiffness was compromised significantly at 3 days. Further, maximal stress was compromised. Actinonin improved these biomechanical parameters. It should be noted that injury-induced recovery of the biomechanical properties was more variable at 7d and that actinonin treatment was not statistically different compared with injury alone at this time point. This may be due to the lack of information regarding the pharmocokinetics of locally injected actinonin, which may decrease in concentration as a function of time after injection. In this study, we did not investigate long-term outcomes with injury. It is possible that the vagina would have fully recovered with injury. In this species, in general, wounds heal by contraction rather than through proliferation of the epithelium and dermal fibroblasts. Further, the animal is not exposed to gravitational forces of bipedal species. Nonetheless, failure of short-term healing in this injury model provides scientific evidence that injury to the vaginal wall may prolong healing and render humans more susceptible to prolapse late in life as sarcopenia of aging may influence compensatory mechanisms of pelvic musculature that support the pelvic organs.

### Actinonin

Remodeling of connective tissue is defined by synthesis and degradation of matrix components. Proteases play an important role in clearing apoptotic cells, fragmented matrix, and damaged cells. Thus, to prepare and synthesis a new matricellular network, proteases play an essential role. Excessive proteolytic damage, however, may tip the balance of successful remodeling to long-term reduced or impaired matrix support. Although elastic fibers are uniquely stable components of most connective tissues, proteolytic damage through UV radiation or cigarette smoke leads to chronic degradation of both elastin and collagen. The proinflammatory effect of elastin degradative peptides accelerates inflammation and impedes wound healing^24^. In this work, elastic fibers were stable after normal vaginal delivery but required regeneration after vaginal injury. This regenerative potential of elastic fibers is unique to the female reproductive tract^25,26^. Actinonin improved postpartum FBLN5 levels modestly, but exhibited a dramatic early effect on injury-induced compromise of biomechanical function. Further studies are thereby warranted regarding the dose, whether continuous treatment is therapeutic or not, and the effects of transient protease inhibitor treatment on long-term outcomes.

### Summary and perspectives

Taken together, this work provides information regarding recovery of the vaginal wall from parturition with or without injury. The strengths are inclusion of protein regulation, biomechanics, and histomorphology at multiple time points postpartum. Results indicate that FBLN5 protein levels, immune cell infiltration, fibrillary collagens, and biomechanical function of the vaginal wall are all negatively impacted by vaginal injury at the time of delivery. Further, actinonin ameliorated these negative effects of injury, but did not restore FBLN5 or collagen type I and III to nonpregnant levels. Weaknesses of the study include the lack of long-term follow-up in the injury model. Characterization of immune cell subtypes and function will be important to understand trafficking of these cells into the vagina with or without injury. Future studies, therefore, will be designed to study the impact of actinonin on elastic fiber proteases indirectly or directly, identification of the protease networks involved, and the impact of actinonin on immune cell trafficking and function.

## Supplementary information

Supplementalinformation

## References

[1] Schaffer JI, Wai CY, Boreham MK (2005). Etiology of pelvic organ prolapse. Clin Obstet Gynecol.

[2] Kim CM (2007). Risk factors for pelvic organ prolapse. Int J Gynaecol Obstet.

[3] Gyhagen M, Bullarbo M, Nielsen TF, Milsom I (2013). Prevalence and risk factors for pelvic organ prolapse 20 years after childbirth: a national cohort study in singleton primiparae after vaginal or caesarean delivery. BJOG.

[4] Memon H, Handa VL (2012). Pelvic floor disorders following vaginal or cesarean delivery. Current opinion in obstetrics & gynecology.

[5] Handa VL (2011). Pelvic floor disorders 5-10 years after vaginal or cesarean childbirth. Obstetrics and gynecology.

[6] Jelovsek JE (2018). Effect of Uterosacral Ligament Suspension vs Sacrospinous Ligament Fixation With or Without Perioperative Behavioral Therapy for Pelvic Organ Vaginal Prolapse on Surgical Outcomes and Prolapse Symptoms at 5 Years in the OPTIMAL Randomized Clinical Trial. Jama.

[7] Nygaard I (2013). Long-term outcomes following abdominal sacrocolpopexy for pelvic organ prolapse. JAMA.

[8] Drewes PG (2007). Pelvic Organ Prolapse in Fibulin-5 Knockout Mice: Pregnancy-Induced Changes in Elastic Fiber Homeostasis in Mouse Vagina. The American Journal of Pathology.

[9] Yanagisawa H (2002). Fibulin-5 is an elastin-binding protein essential for elastic fibre development *in vivo*. Nature.

[10] Nakamura T (2002). Fibulin-5/DANCE is essential for elastogenesis *in vivo*. Nature.

[11] Budatha M (2011). Extracellular matrix proteases contribute to progression of pelvic organ prolapse in mice and humans. J Clin Invest.

[12] Budatha M (2013). Dysregulation of protease and protease inhibitors in a mouse model of human pelvic organ prolapse. Plos one.

[13] Rahn DD, Ruff MD, Brown SA, Tibbals HF, Word RA (2008). Biomechanical properties of the vaginal wall: effect of pregnancy, elastic fiber deficiency, and pelvic organ prolapse. Am J Obstet Gynecol.

[14] Chin K (2016). Pelvic Organ Support in Animals with Partial Loss of Fibulin-5 in the Vaginal Wall. Plos one.

[15] Florian-Rodriguez M (2019). Effect of Protease Inhibitors in Healing of the Vaginal Wall. Sci Rep.

[16] Delancey JO (2002). Fascial and muscular abnormalities in women with urethral hypermobility and anterior vaginal wall prolapse. Am J Obstet Gynecol.

[17] Richardson AC, Edmonds PB, Williams NL (1981). Treatment of stress urinary incontinence due to paravaginal fascial defect. Obstet Gynecol.

[18] Liu X (2004). Elastic fiber homeostasis requires lysyl oxidase-like 1 protein. Nat Genet.

[19] Rahn DD (2009). Failure of pelvic organ support in mice deficient in fibulin-3. Am J Pathol.

[20] Downing KT (2014). The role of mode of delivery on elastic fiber architecture and vaginal vault elasticity: a rodent model study. J Mech Behav Biomed Mater.

[21] Nygaard IE (2017). Physical and cultural determinants of postpartum pelvic floor support and symptoms following vaginal delivery: a protocol for a mixed-methods prospective cohort study. BMJ Open.

[22] Lowder JL (2007). Biomechanical adaptations of the rat vagina and supportive tissues in pregnancy to accommodate delivery. Obstetrics and gynecology.

[23] Feola A (2011). Impact of pregnancy and vaginal delivery on the passive and active mechanics of the rat vagina. Annals of biomedical engineering.

[24] Almine JF (2013). Elastin sequences trigger transient proinflammatory responses by human dermal fibroblasts. FASEB J.

[25] Starcher B, Percival S (1985). Elastin turnover in the rat uterus. Connective tissue research.

[26] Fata JE, Ho AT, Leco KJ, Moorehead RA, Khokha R (2000). Cellular turnover and extracellular matrix remodeling in female reproductive tissues: functions of metalloproteinases and their inhibitors. Cell Mol Life Sci.

