# Supplementary material for: Protease Inhibition Improves Healing of The Vaginal Wall after Obstetrical Injury: Results from a Preclinical Animal Model
Source: Sci Rep. 2020 Apr 14;10:6358. doi: 10.1038/s41598-020-63031-6 (PMC7156712; doi:10.1038/s41598-020-63031-6)
Supplement: Supplementary file 1 — Supplementalinformation [file 41598_2020_63031_MOESM1_ESM.pdf]

PROTEASE INHIBITION IMPROVES HEALING OF THE VAGINAL WALL AFTER OBSTETRICAL INJURY: RESULTS FROM A PRECLINICAL ANIMAL MODEL

Jennifer Hamner DO[1], Maria Florian-Rodriguez MD[2], Jesus Acevedo MS[2], Haolin Shi[2], and R Ann Word MD[2]

[1]Department of Obstetrics and Gynecology[1]
Division of Female Pelvic Medicine and Reconstructive Surgery
University of Texas Southwestern Medical Center, Dallas, Texas, USA
[2]Department of Obstetrics and Gynecology
Cecil H and Ida Green Center for Reproductive Biological Sciences
Division of Female Pelvic Medicine and Reconstructive Surgery
University of Texas Southwestern Medical Center, Dallas, Texas, USA

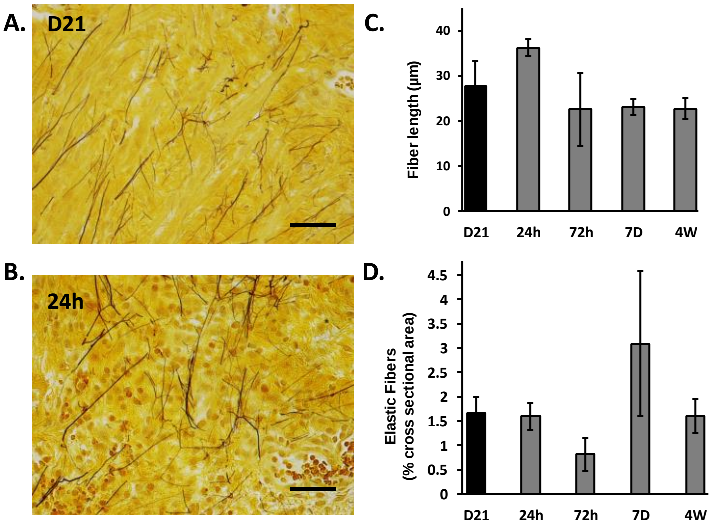

Supplementary Figure 1. Elastic fiber morphology and quantification after normal vaginal delivery. Hart's staining revealed long elastic fibers throughout the vaginal muscularis of the posterior vaginal wall in pregnant rats in late gestation (D21, **A**) which were similar 24h after delivery despite immune cell infiltration **(B)**. **(C)** Vaginal elastic fiber length during pregnancy and postpartum. **(D)** Cross sectional area covered by elastic fibers in the vaginal muscularis during pregnancy and the postpartum time period. Results represent mean ± SEM of 5 animals in each group. Bar, 50 µm

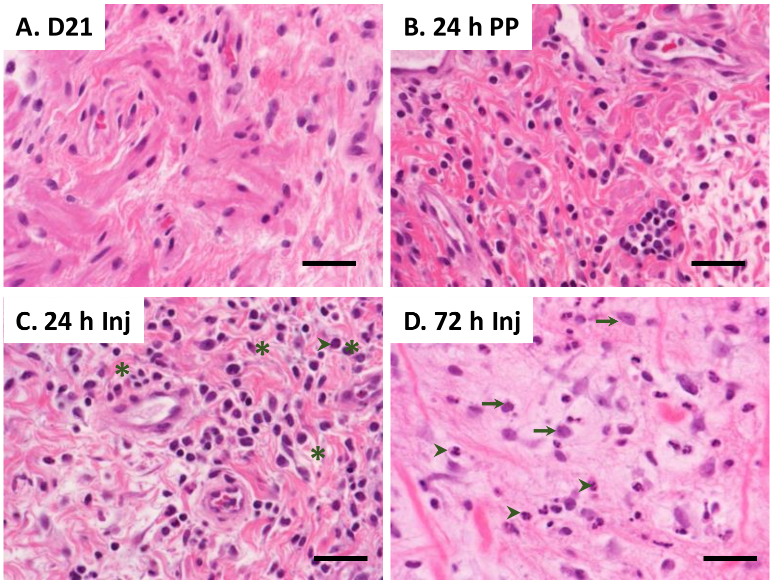

Supplementary Figure 2. Effect of vaginal delivery with or without injury on immune cells in the parturient vagina. H&E staining revealed few resident immune cells on D21 of pregnancy **(A)**. After normal vaginal delivery (24h, **B**) monocytes and neutrophils were localized within blood vessels and infiltrated the muscularis. After injury (24h, **C**), monocytes and neutrophils (arrowhead) were abundant and surrounded by a pericellular halo (*) and matrix degradation. By 3d **(D)**, immune cells included neutrophils (arrowheads) and macrophages (arrows). Bar, 50 µm

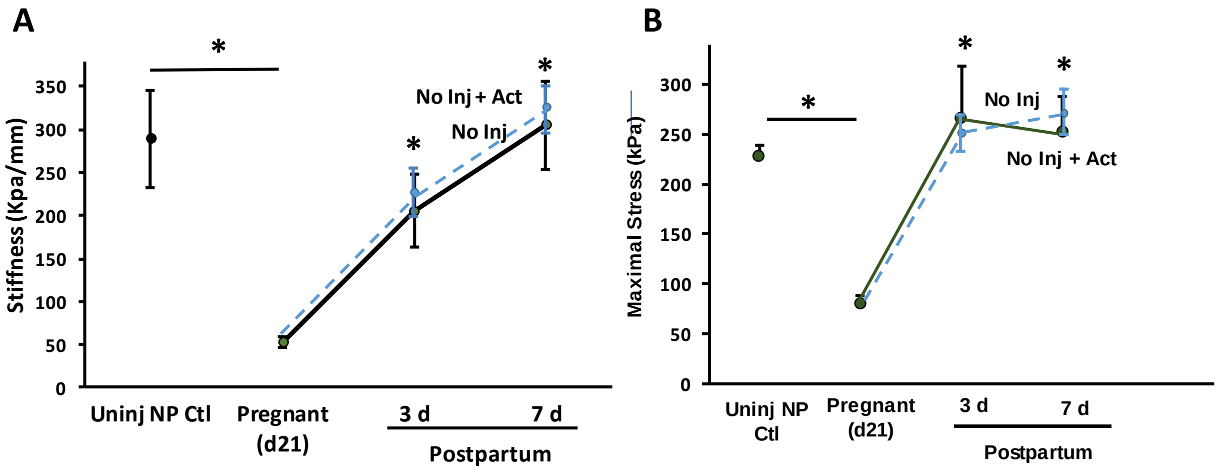

Supplementary Figure 3. Effect of actinonin on biomechanical properties of the postpartum uninjured rat vaginal wall. (A) Stiffness was quantified from the slope of the linear portion of stress-strain curve. Postpartum time points of 3 and 7d with or without actinonin (Act) are represented (no injury). **(B)** Maximal stress generating capacity in nonpregnant, pregnant, and postpartum animals without injury with or without actinonin. Data represent mean ± SEM of nonpregnant (n=8), normal postpartum with (n = 4 per 3 D and 7 D time points, dashed line)or without actinonin (n = 8 per time point, solid line). *, P < NP or P (d21). Actinonin alone had no effect on normal postpartum recovery
